# Role of Sinonasal Anatomic Variations in the Development of Maxillary Sinusitis: A Cone Beam CT Analysis

**DOI:** 10.2174/1874210601711010367

**Published:** 2017-06-30

**Authors:** Leila Khojastepour, Abdolaziz Haghnegahdar, Negar Khosravifard

**Affiliations:** 1 Department of Oral and Maxillofacial Radiology, School of Dentistry, Shiraz University of Medical sciences, Shiraz, Iran.; 2 Department of Oral and Maxillofacial Radiology, School of Dentistry, Guilan University of Medical sciences, Rasht, Iran.

**Keywords:** Haller cells, Uncinate process, Maxillary sinus ostium, Maxillary sinusitis, Cone beam CT

## Abstract

**Background:**

Several anatomical variations can lead to the inflammation of the paranasal sinuses; therefore, surgeons should be familiar with these variations and their impacts on the status of the paranasal sinuses.

**Objectives:**

The present study aimed to determine the prevalence of Haller cells and its association with patients’ sex and age. Furthermore, the relationships between the presence and size of Haller cells, deviation of the uncinate process and size of the maxillary sinus ostium with the occurrence of maxillary sinusitis were investigated.

**Materials/ Patients and Methods:**

120 coronal CBCT images were retrieved and analyzed. Statistical analysis of the data was performed by means of Mann - Whitney, χ^2^ and T tests.

**Results:**

There were statistically significant associations between the presence and surface area of Haller cells and the occurrence of ipsilateral maxillary sinusitis. Neither the angulation of the uncinate process nor the size of the maxillary sinus ostium significantly correlates with the formation of maxillary sinusitis.

**Conclusion:**

Haller cells can interfere with the normal drainage of the maxillary sinus and result in sinusitis. In contrast, diameter of the sinus ostium and deviation of the uncinate process do not influence the inflammatory status of the maxillary sinus significantly.

## BACKGROUND

Anatomical variations of the paranasal sinuses can cause various sinus pathologies and complicate sinus surgeries. Deviation of the nasal septum, concha bullosa, agger nasi cells, lateral or medial bending of the uncinate process and Haller cells are some of the aforementioned variations [1].

Haller cells were first introduced by Albrecht Von Haller in 1756 and were named after him; however, the terminology for these air cells has been changed to infraorbital ethmoid cells, as they originate from the anterior ethmoid cells and are located in the medial orbital floor [2].

Haller cells are frequently seen as incidental findings in CT examinations of paranasal sinuses. The location of Haller cells in the medial portion of the orbital floor and lateral to the maxillary infundibulum makes the pattern of mucociliary flow prone to deterioration and, therefore, increases the risk of recurrent maxillary sinusitis [3]. The incidence of Haller cells reported in different studies covers a wide range from 2%-70.3%. [2-8].

In the study performed by Alkire *et al*, the authors compared the impact of septal deviation, concha bullosa and Haller cells on the occurrence of rhinosinusitis. They concluded that only the obstruction caused by Haller cells was statistically relevant with the development of this pathologic condition [8]. Furthermore, several radiographic studies have shown a significant relationship between Haller cells measuring larger than 3 mm and maxillary sinusitis [9-11].

The presence of infraorbital ethmoidal (Haller) cells can increase the risk of orbital injury during ethmoidectomy [12]. Functional endoscopic sinus surgery is the primary approach used today for the surgical treatment of chronic sinusitis. Diagnosis of Haller cells, however, is difficult in endoscopy due to their lateral location [13] . Failure to recognize Haller cell also can increase the risk of orbital injury during ethmoidectomy [12].

Maxillary sinus ostium is closely related to the nasolacrimal canal, ethmoidal infundibulum and orbit floor [14]. Variation in the infraorbital (antero-supero-medial) angle of the sinus needs more attention [15].

Hammad *et al.* evaluated the role of some anatomical nasal abnormalities (deviated nasal septum, concha bullosa and Haller cell) in rhinogenic headache,vacuum headache, and pressure headache. They proposed that as a main cause of referred headache, Haller cell can occupy the infundibulum or be associated with another anatomical variation, such as concha bullosa, and can predispose to sinusitis. In the absence of sinusitis, it might block the sinus drainage pathway, resulting in sinus malventilation, vacuum headache, and pressure headache [16].

Haller’s cells also can reach the infraorbital nerve. Choi [13] recently reported a case of Hypoesthesia of midrace by isolated Haller’s cell mucocele and stated that although isolated infection of the Haller’s cell is usually very rare, it should be suspected in patients with facial pain and hypoesthesia [13].

According to the previous researches, both spatial resolution and soft tissue contrast of CBCT are sufficient to aid in surgical navigation of the sinonasal cavities. Moreover, patients are exposed to lower amounts of radiation during CBCT examination compared with multislice CT [17-19].

### OBJECTIVES

The present study was performed based on data gathered from CBCT images of an Iranian population and aimed at evaluating the correlation between maxillary sinusitis and several anatomical variations including the presence and surface area of Haller cells, angulation of the uncinate process and size of the maxillary sinus ostium.

### MATERIALS/ PATIENTS AND METHODS

Considering exclusion criteria, 120 CBCT images from the archives of an oral and maxillofacial radiology center were used as samples for the present study. All CBCTs were taken as part of preoperative recording of patients seeking rhinoplasty in an otolaryngology clinic over a 1-year period.

History of sinus tumor or surgery, sinonasal polyposis and maxillofacial trauma was considered as exclusion criteria. As errors in patient positioning could possibly result in inaccurate measurements, images with faulty patient orientations (such as head tilt) were excluded. All CBCT images were acquired by NewTom VGi (NewTom GR srl-verona, Italy) with following specifications: scan time of 36 s, field of view of 15×12 cm, 110 kVp and 20 mA. The images were viewed by NNT software in coronal cross-sections as the ostiomeatal complex is most reliably evaluated in the coronal plane [1, 8]. Since the CBCT records had been previously prescribed for other medical purposes, no extra radiation was imposed on the patients. Personal information of all individuals was kept totally undisclosed; hence, the study did not face any ethical limitations and was approved by the Ethical Committee of Shiraz University of Medical Sciences

The following items were evaluated on each CBCT image:

- Presence of Haller cells
- Dimensions of the Haller cells in both vertical and horizontal orientations
- Medial or lateral deviation of the uncinate process
- Size of the ostia of maxillary sinuses
- Presence of mucosal thickening in the maxillary sinuses

**Fig. (2):**
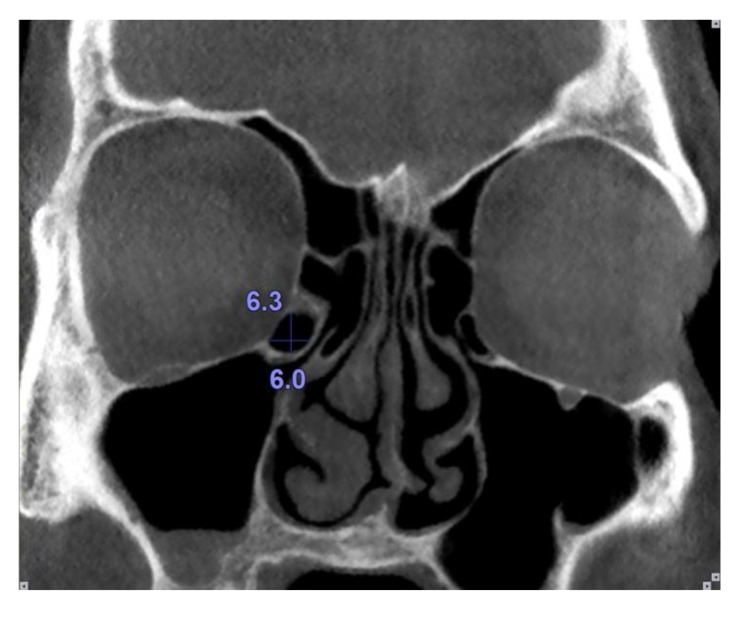
Maximum vertical and horizontal dimensions of the Haller cells were measured.

Haller cells were considered as air cells located along the medial portion of the orbital floor and continuous with the ethmoid capsule Fig. (**1**). Size of the Haller cells was measured at the maximum superoinferior and mediolateral dimensions (Fig. **2**).

**Fig. (3):**
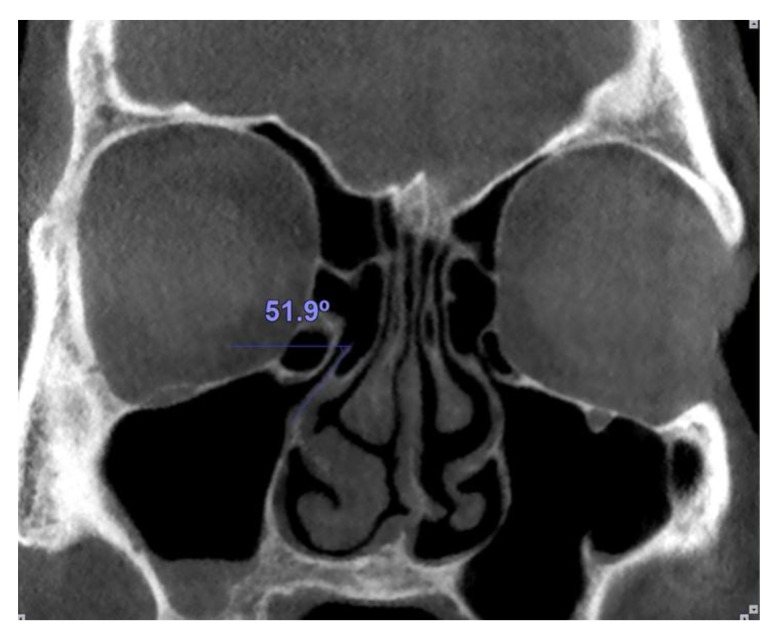
The angle between the uncinate process and a horizontal line passing from its superior edge was measured.

Medial or lateral deviation of the uncinate process was determined by measuring the angle of the process with a horizontal line passing from its superior edge (Fig. **3**).

**Fig. (4):**
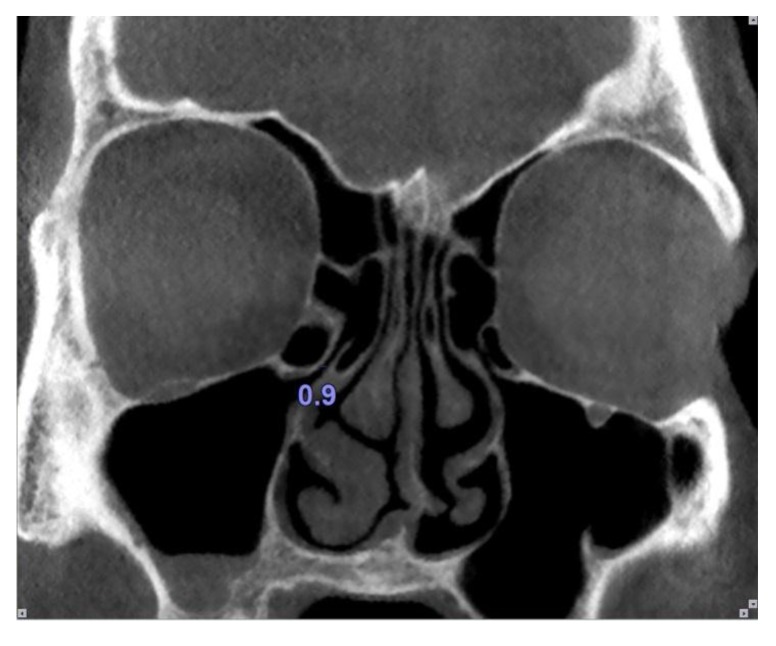
Size of the maxillary sinus ostium was measured as the distance between the Haller cell and the uncinate process.

The maxillary sinus ostium was defined as the distance between the inferomedial aspect of the orbital rim and the uncinate process. Obviously, in the cases where Haller cells existed, the maxillary sinus ostium was quantified as the distance between the Haller cell at its most medial portion and the uncinate process (Fig. **4**).

All distance and angular measurements were done by an expert oral radiologist with more than 20 years of experience using NNT software version 6.2 (NewTom GR srl-verona, Italy) measuring tools at two fold magnification (200%). For statistical analysis, the T- test was used for the evaluation of the following associations:

- Patients’ age and presence of Haller cells
- Angulation of the uncinate process and mucosal thickening of the maxillary sinus
- Size of the maxillary sinus ostium and mucosal thickening of the maxillary sinus

The χ^2^ test evaluated the associations between:

- Patients’ sex and presence of Haller cells
- Presence of Haller cells and mucosal thickening of the maxillary sinus

The Mann-Whitney test was utilized to evaluate the association between surface area of the Haller cells and mucosal thickening of the maxillary sinus. *P* < 0.05 was considered to be statistically significant.

## RESULTS

Among 120 patients included in the investigation, 57 (47.5%) were females and 63 (52.5%) were males with ages ranging from 9 to 57 years (mean 27.78 ± 9.93 years). Haller cells were detected in 50 patients (41.6%); out of which, 36 (30%) were unilateral and 14 (11.6%) were bilateral. Among the 36 unilateral Haller cells, 29 (80.5%) were right sided and 7 (19.5%) were left sided. Both vertical and horizontal dimensions of the Haller cells were measured and the surface area of each Haller cell was calculated as an elliptical area (Table **1**).

As presented in Table (**2**), the associations of both the presence and surface area of Haller cells with mucosal thickening in the ipsilateral maxillary sinus were statistically significant; contrarily, there were no statistically significant associations between patients’ sex and presence of Haller cells, patients’ age and presence of Haller cells, size of the maxillary sinus ostium and presence of mucosal thickening in the sinus and angulation of the uncinate process and mucosal thickening of the maxillary sinus.

## DISCUSSION

The incidence of Haller cells reported in different studies covers a wide range from 5.5% to 45.9% [2]. This diversity among different studies could be explained by various factors such as the patients’ age and race, imaging techniques used and the exact definition of Haller cells by different authors [3, 20]. For instance, Bolger *et al.* defined Haller cell as any air cell located between the ethmoid bulla, the orbital lamina of the ethmoid bone and the orbital floor [9]. In the study performed by Kennedy and Zinreich, Haller cells were considered as ethmoid cells located below the ethmoid bulla within the orbital floor and in the region of the opening of the maxillary sinus [21]. Furthermore, Kainz *et al.* defined Haller cells as air cells within the orbital floor [10].

In the present study, the criteria that we used for the definition of Haller cells were similar to the study performed by Mathew *et al, i.e*., air cells located along the medial portion of the orbital floor and/or the lamina papyracea inferior to the bulla ethmoidalis and continuous with the ethmoid capsule were considered as Haller cells [3]. The frequency of Haller cells in our study was determined at 41.6% which is almost equal to the prevalence measured by Pekiner *et al,* [22]. This finding indicates the accuracy of CBCT in detecting these cells regardless of their size which is not a feature of multislice CT due to the interslice missing of small air cells .

Based on our investigation, neither patients’ sex nor their age had significant correlations with the presence of Haller cells; however, both the presence and size of Haller cells revealed significant associations with the mucosal thickening in the ipsilateral maxillary sinus. This could be explained by the critical location of Haller cells immediately lateral to the maxillary infundibulum which can interfere with the normal drainage of the maxillary sinus. Several investigators have also come to the same conclusion regarding the association between the presence of Haller cells and maxillary sinusitis [23-25]; on the other hand, some authors believe that only medium and large sized Haller cells can lead to mucosal thickening of the maxillary sinus [26, 27].

Based on the present study, the association between mucosal thickening of the maxillary sinus and size of the sinus ostium was not statistically significant, which is in conformity with the study results of Mathew *et al,* [3]. This finding suggests that maxillary sinusitis is likely to be a primary condition rather than a condition originating from mechanical obstruction of the sinus ostium.

Our study did not reveal a significant association between deviation of the uncinate process and mucosal thickening in the maxillary sinus which could be explained by taking into account that narrowing or obstruction of the ostium that could not be considered as the primary causes of maxillary sinusitis.

## CONCLUSION

In conclusion, our study disclosed significant association between the presence and surface area of Haller cells with maxillary sinusitis. On the contrary, the size of maxillary sinus ostium and deviation of the uncinate process were not significantly associated with maxillary sinusitis. Patients’ age and sex had no influence on the prevalence of Haller cells. Moreover, it was concluded that CBCT has the capability to demonstrate fine anatomic details of the ostiomeatal complex at far less radiation amounts and, therefore, is recommended as a suitable imaging technique for the evaluation of sinonasal cavities.

## Figures and Tables

**Fig. (1):**
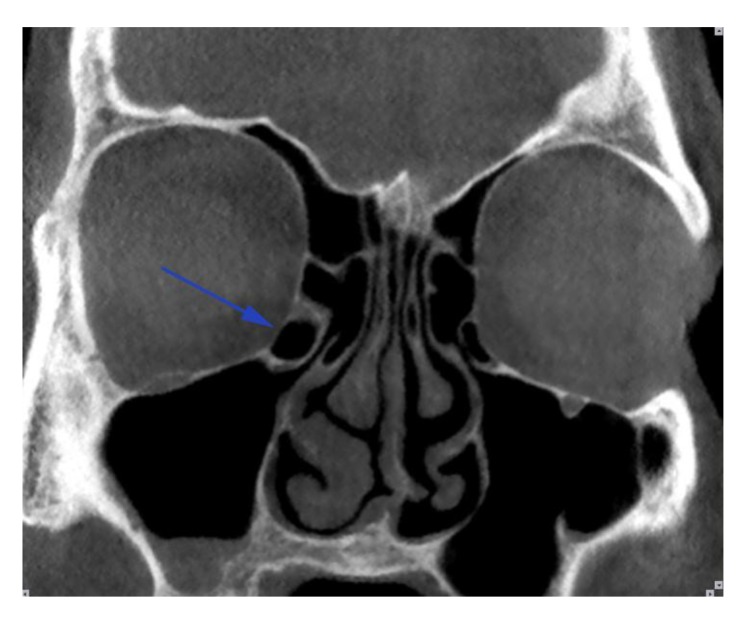
Arrow points to a Haller cell in the right side.

**Table 1:** Minimum, maximum and mean ± SD values of the surface areas of right and left Haller cells.

	Right Haller Cells	Left Haller Cells
Minimum surface area Maximum surface area Mean ± SD of surface area	8.48 77.15 30 ± 21.35	3.63 39 18.06 ± 10.29

**Table 2:** Summary of the associations between variables evaluated in the investigation.

Evaluated Variables	Statistical Analysis	*P*- value	Interpretation (Association)
Patients’ sex and presence of Haller cells	χ^2^ test	*P*=0.233	No significant
Patients’ age and presence of Haller cells	T test	*P*=0.879	No significant
Presence of Haller cells and mucosal thickening of the maxillary sinus on the right side	χ^2^ test	*P*=0.037	Significant
Presence of Haller cells and mucosal thickening of the maxillary sinus on the left side	χ^2^ test	*P*=0.032	Significant
Surface area of Haller cells and mucosal thickening of the maxillary sinus on the right side	Mann-Whitney test	*P*=0.045	Significant
Surface area of Haller cells and mucosal thickening of the maxillary sinus on the left side	Mann-Whitney test	*P*=0.038	Significant
Size of maxillary sinus ostium and mucosal thickening on the right side	T test	*P*=0.145	No significant
Size of maxillary sinus ostium and mucosal thickening on the left side	T test	*P*=0.220	No significant
Angulation of the uncinate process and mucosal thickening of the maxillary sinus on the right side	T test	*P*=0.708	No significant
Angulation of the uncinate process and mucosal thickening of the maxillary sinus on the left side	T test	*P*=0.589	No significant
